# Fast optimization of non-negative matrix tri-factorization

**DOI:** 10.1371/journal.pone.0217994

**Published:** 2019-06-11

**Authors:** Andrej Čopar, Blaž Zupan, Marinka Zitnik

**Affiliations:** 1 Faculty of Computer and Information Science, University of Ljubljana, Ljubljana, Slovenia; 2 Department of Molecular and Human Genetics, Baylor College of Medicine, Houston, TX, United States of America; 3 Department of Computer Science, Stanford University, Stanford, CA, United States of America; University of Bonn, Bonn-Aachen International Center for IT, GERMANY

## Abstract

Non-negative matrix tri-factorization (NMTF) is a popular technique for learning low-dimensional feature representation of relational data. Currently, NMTF learns a representation of a dataset through an optimization procedure that typically uses multiplicative update rules. This procedure has had limited success, and its failure cases have not been well understood. We here perform an empirical study involving six large datasets comparing multiplicative update rules with three alternative optimization methods, including alternating least squares, projected gradients, and coordinate descent. We find that methods based on projected gradients and coordinate descent converge up to twenty-four times faster than multiplicative update rules. Furthermore, alternating least squares method can quickly train NMTF models on sparse datasets but often fails on dense datasets. Coordinate descent-based NMTF converges up to sixteen times faster compared to well-established methods.

## Introduction

Extracting patterns from relational data is a key task in natural language processing [1], bioinformatics [2], and digital humanities [3]. We typically represent a relational dataset with a data matrix, encoding, for example, information on document-term frequencies, gene-disease associations, or user-item ratings. Non-negative matrix tri-factorization (NMTF) is a general technique that takes a data matrix and compresses, or embeds, the matrix into a compact latent space. The learned embedding space can be used to identify clusters [4, 5], reveal interesting patterns [6, 7], and generate feature representations for downstream analytics [8, 9]. NMTF has been used to discover disease-disease associations [10]. identify cancer driver genes from patient data [11], and to model topics in text data [12]. However, despite numerous applications, training NMTF models on large datasets can be slow and has remained computationally challenging [13].

**Fig 1:**
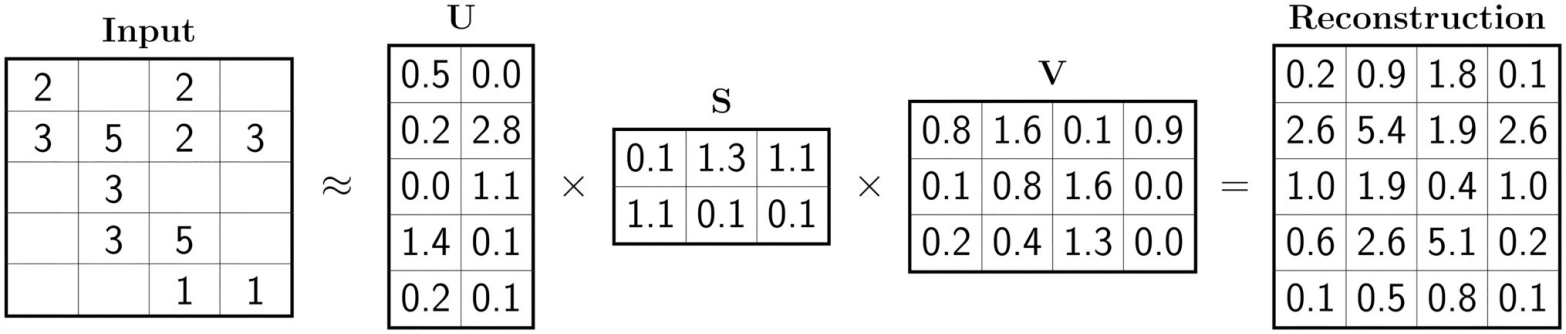
Non-negative matrix tri-factorization (NMTF). An input 5 × 4 sparse data matrix (left) is approximated by a product of three non-negative low-dimensional latent matrices (**U**, **S**, and **V**). The product (right) gives a reconstruction of the original data matrix. The reconstructed matrix has all of its elements completed, which can be leveraged for prediction. The goal of training an NMTF model is to find the latent matrices that produce a high-quality reconstruction of the input matrix [28].

A distinguishing property of non-negative matrix tri-factorization is that it factorizes a given data matrix and represents it with a product of three non-negative low-dimensional matrices, often called latent matrices [14] (Fig 1). While these latent matrices are key to matrix tri-factorization, finding the factorization of a given matrix is an NP-hard problem [15]. We thus use optimization methods to find latent matrices that approximately factorize the matrix. A traditional approach uses multiplicative update rules [4], a method, which iteratively revises latent matrices to minimize the approximation error. Such an iterative update involves multiplying the current approximation with the gradient of the objective function, which captures the discrepancy between the input data matrix and its latent-based reconstruction. Several studies improved the performance of multiplicative update rules, for example, by using parallelization [16, 17]. A significant limitation of multiplicative update rules is that the method is slow to converge [13]. For this reason, classic non-negative matrix factorization [18] has been studied using alternative training algorithms, including alternating least squares [19, 20], projected gradients [21, 22], and coordinate descent [20]; however, these methods have not been investigated for non-negative matrix tri-factorization.

Here, we develop three training algorithms for non-negative matrix tri-factorization that use projected gradients, coordinate descent, and alternating least squares optimization. First, projected gradient method uses a step-size parameter to maximize the learning rate without compromising non-negative constraints on latent matrices in NMTF [21]. Second, coordinate descent method uses partial computation result of latent matrices to successively adjust the update step, decomposing the update of latent matrices into a series of coordinate-specific, or latent factor-specific, updates. It has been shown that coordinate descent can reduce the computational time of machine learning methods, such as support vector machines [23] and classic non-negative matrix factorization [20, 24]. Third, alternating least squares method alternates between updating one latent matrix while fixing the other two [19]. The success of these three methods for various tasks in machine learning [25–27] encouraged us to adapt them for non-negative matrix tri-factorization. We derive projected gradients, coordinate descent, and alternating least squares methods for NMTF. We then show convergence and runtime improvements of the new training algorithms over traditional multiplicative update rules on six datasets.

## Materials and methods

We first describe datasets and their preprocessing. We then continue with a formal presentation of optimization methods, focusing on derivation of three optimization methods that are new for non-negative matrix tri-factorization.

### Datasets and preprocessing

We considered six datasets of varying size and density (Table 1). These datasets are popular benchmark datasets in the analysis of relational data and matrix factorization. (1) AlphaDigits [29] is a binary dataset of 1404 hand-drawn images of numbers and letters with dimensions of 16x20. (2) Coil20 [30] is a dataset of 1440 images each of size 128x128. Images from both datasets were flattened into a single 16,384-column vector and each pixel is represented with a value in range 0-255. (3) Mutations [31] contains a sparse binary matrix of almost five thousand patient samples with 19 different types of tumors and somatic mutations in 25 thousand genes. (4) MovieLens [32] is a sparse dataset of 10 million ratings given to ten thousand movies from 70 thousand different users. Each rating is represented by a discrete value between 0 and 5. (5) Newsgroups [33] is a real-valued sparse document-term dataset containing over 10 thousand documents with 73 thousand terms. Stop words are removed from the text and TF-IDF is used to generate feature vectors. (6) Finally, STRING dataset [34] contains binary and undirected protein-protein interaction network for Homo Sapiens, which we obtain from the STRING database.

**Table 1:** Datasets considered in this study. ‘Nonzero’ indicates the number of non-zero values in a data matrix.

Dataset	Rows	Columns	Density (%)	Non-zero
AlphaDigits	1404	320	100.0	0.45M
Coil20	1440	16,384	100.0	23M
STRING	19,576	19,576	2.9	11.3M
MovieLens	69,878	10,677	1.3	9.7M
Mutations	4,790	25,169	0.8	1M
Newsgroups	18,821	70,066	0.1	1.4M

### Non-negative matrix tri-factorization (NMTF)

Non-negative matrix tri-factorization (NMTF) aims to represent the data $X\in R^{n\times m}$ with a product of three non-negative latent matrices $U\in R_{+}^{n\times k_{1}}$, $S\in R_{+}^{k_{1}\times k_{2}}$ and $V\in R_{+}^{m\times k_{2}}$ [4]. Here, parameters *k*_1_ and *k*_2_ represent factorization ranks and describe the number of latent vectors that form the row and column space, respectively. Matrix factorization can reduce dimensionality and noise of the original input data matrix **X** [35], and provide an understanding of the latent structure present in the data. In contrast to classic non-negative matrix factorization [18], which decomposes the input matrix into two latent matrices, NMTF decomposes the input matrix into three latent matrices. Here, latent matrix **U** approximates the row vector space of **X** with a *k*_1_-dimensional vector space. Similarly, **V** describes a column-space with *k*_2_ vectors, and **S** describes interactions between the two latent vector spaces.

Discrepancy between input data matrix **X** and its reconstruction $\hat{X}=USV^{T}$ is measured through a loss function that aims to minimize the following Frobenius distance *D*_Fro_:

$$\begin{array}{c} D_{\text{Fro}}(X||USV^{T})=||X-USV^{T}{\left|\right|}_{\text{Fro}}^{2}. \end{array}$$ (1)

Here, several alternative loss functions can be used, such as the Kullback-Leibler divergence, Alpha divergence [36], and Beta divergence [37]. In addition to non-negativity, we can promote other structural properties by including additional regularization terms in the loss function *D*_Fro_. In particular, in clustering applications, we might want to impose orthogonality on **U** and **V** [4] such that **U**’s and **V**’s latent vectors indicate memberships of row and column objects in distinct clusters [38]. For example, adding **U**^*T*^ **U** regularization term to the loss function will make latent vectors in **U** to be orthogonal to each other. Another popular approach is to impose sparsity constraints on latent matrices by including ||**U**||_1_ and ||**V**||_1_ regularization in the loss function [39]. In this paper, we develop optimization algorithms, which optimize an objective function that consists of the reconstruction error and does not include any additional constraints or regularization terms other than non-negativity of latent matrices.

### Multiplicative update rules for NMTF

The objective function of NMTF is non-convex; however when we fix all but one latent matrix, the function becomes convex [4]. Minimization of the objective function with respect to each of the three latent matrices **U**, **V** and **S**, allows the algorithm to converge to a local stationary point [13]. Multiplicative update rules start by initializing latent matrices with random values and then iteratively update the matrices in the direction of the gradient until convergence. Convergence criteria is often measured as difference in the value of objective function in Eq 1 between two or more successive iterations of the algorithm. Next, we give a summary derivation of existing multiplicative update rules [28]. Karush Kuhn-Tucker condition $\frac{\partial F}{\partial UU_{ik}}=0$ takes the partial derivative of **U** and calculates the updated **U** matrix at *i*-th row and *k*-th column. The resulting update rule for **U** is as follows:

$$\begin{array}{c} U\leftarrow U⊙({XVS}^{T}⊘{USV}^{T}{VS}^{T}), \end{array}$$ (2)

where symbol ⊙ denotes Hadamard product and symbol ⊘ denotes Hadamard division. Similarly, the update rule for **V** is derived:

$$\begin{array}{c} V\leftarrow V⊙(X^{T}US⊘{VS}^{T}U^{T}US). \end{array}$$ (3)

Finally, to obtain the update rule for latent matrix **S**, we take derivative of the objective function with respect to **S** and use the Karush Kuhn-Tucker conditions for **S**. This procedure gives the following update rule for **S**:

$$\begin{array}{c} S\leftarrow S⊙(U^{T}XV⊘U^{T}{USV}^{T}V). \end{array}$$ (4)

Section A in S1 File shows derivations of multiplicative update rules.

### Alternating least squares for NMTF

Alternating least squares method [40] iteratively updates the latent matrices and each update involves solving a least-squares problem. Here, we obtain the update rules by deriving the objective function in Eq 1 for each latent matrix and then enforcing non-negativity on the latent matrix using a heuristic. This derivation procedure gives the following update rules:

$$\begin{array}{ccc} U & \leftarrow & [\left({XVS}^{T}\right){\left({SV}^{T}{VS}^{T}\right)}^{-1}{]}_{+},\\ V & \leftarrow & [\left(X^{T}US\right){\left(S^{T}U^{T}US\right)}^{-1}{]}_{+},\\ S & \leftarrow & [{\left(U^{T}U\right)}^{-1}\left(U^{T}XV\right){\left(V^{T}V\right)}^{-1}{]}_{+}, \end{array}$$ (5)

where [**A**]_+_ is projection to non-negative space, calculated as **A**_*ij*_ = 0 if **A**_*ij*_ < 0 else **A**_*ij*_. Alternating least squares approach is equivalent to the second-order quasi-Newton approach [5]. Section B in S1 File shows full derivations of alternating least squares approach, where Section E in S1 File shows the derivation of quasi-Newton approach and its equivalence to alternating least squares. Efficient implementations of alternating least squares method is as fast as multiplicative update rules but has unstable convergence. This is because alternating least squares method transforms current approximation of the latent matrices into non-negative matrices by simply replacing all negative values with zero values [19].

### Projected gradients for NMTF

Optimization of matrix factorization models that use gradient descent [18] repeatedly apply additive updates to model parameters in the direction specified by the gradient of the objective function and using a particular step size. The selection of the step size is not trivial [41]. When using a large fixed step size, we risk accidentally increasing the value of objective function. When the step size is too small, it can significantly slow down the convergence speed.

Projected gradients method is a gradient-based optimization method intended for solving constrained convex problems [42]. In the case of non-negative matrix tri-factorization, the method realizes the non-negativity constraints by projecting negative values in a latent matrix to a non-negative space [43]. The method is similar to multiplicative update rules. In particular, it uses an adaptive learning rate (*i.e*., step-size parameter) that is automatically determined in order to perform a maximum possible step in the gradient direction while staying in the non-negative space. In contrast to alternating least squares, projected gradients method is able to handle the non-negativity constraint of latent matrices in a more principled way [44]. Note that by setting the step-size parameter to 1, the update rules become equivalent to multiplicative update rule.

We derive projected gradients for NMTF and obtain the following update rule for latent matrix **U**:

$$\begin{array}{ccc} P_{u} & = & U-U⊘\left({USV}^{T}{VS}^{T}\right)⊙\left({XVS}^{T}\right),\\ \eta_{u} & = & \frac{\sum (P_{u}⊙\left(USV^{T}VS^{T}-XVS^{T}\right))}{Tr(\left(SV^{T}V\right)\left(S^{T}{P_{u}}^{T}P_{u}\right))},\\ U & \leftarrow & [U-\eta_{u}P_{u}{]}_{+}, \end{array}$$ (6)

where **P_u_** is a projection matrix, and *η*_*u*_ is step-size parameter. The update rule for latent matrix **V** is as follows:

$$\begin{array}{ccc} P_{v} & = & \text{V}-\text{V}⊘(VS^{T}{\text{U}}^{T}US)⊙(X^{T}US),\\ \eta_{v} & = & \frac{\sum \left(P_{v}⊙\left(VS^{T}U^{T}US-X^{T}US\right)\right)}{Tr\left(\left(SP_{v}{}^{T}P_{v}\right)\left(S^{T}U^{T}U\right)\right)},\\ V & \leftarrow & {\left[V-\eta_{v}P_{v}\right]}_{+}, \end{array}$$ (7)

where **P_v_** is a projection matrix, and *η*_*v*_ is a step-size value. The update rule for latent matrix **S** is as follows:

$$\begin{array}{ccc} P_{s} & = & S-S⊘\left(U^{T}{USV}^{T}V\right)⊙\left(U^{T}XV\right),\\ \eta_{s} & = & \frac{\sum (P_{s}⊙\left(U^{T}{USV}^{T}V-U^{T}XV\right))}{Tr(\left(U^{T}UP_{s}\right)\left(V^{T}V{P_{s}}^{T}\right))},\\ S & \leftarrow & [S-\eta_{s}P_{s}{]}_{+}, \end{array}$$ (8)

where **P_s_** is a projection matrix, and *η*_*s*_ is a step-size value. Section C in S1 File shows full derivations of projected gradient approach.

### Coordinate descent for NMTF

Coordinate descent is an optimization method widely used in machine learning, including in support vector machines [45], and non-negative matrix factorization (NMF) [20, 46]. Coordinate descent has been proposed as an alternative approach for NMF methods, and its advantages for two-factor NMF and multiplicative updates have been already reported [47–49]. In contrast to the multiplicative and gradient-based method, which update latent matrices in a joint gradient direction, coordinate descent separately computes the gradient of each vector in each latent matrix.

Coordinate descent is a first-order method, similar to multiplicative update rules, alternating least squares, and projected gradients. While other methods use derivatives of entire latent matrices, coordinate descent computes derivatives concerning scalars or one-dimensional vectors of latent matrices and re-use partially computed results as soon as possible [50]. For example, updates to the first vector in a latent matrix are included in computing the second one, and the values from the first two vectors are then used to compute the third vector. Coordinate descent can use different ordering of vector updates, which gives rise to different variants of the method [51]: cyclic coordinate descent, stochastic coordinate descent, and greedy coordinate descent. The cyclic approach uses the same ordering of updates in each iteration of the algorithm, whereas a stochastic approach uses a random order of updates. Finally, a greedy approach [49] selects to update the vector that reduce objective function the most.

Next, we present NMTF update rules implementing cyclic coordinate descent:

$$\begin{array}{ccc} u_{\cdot i} & \leftarrow & u_{\cdot i}+[\frac{{\left({XVS}^{T}\right)}_{\cdot i}-{\left({USV}^{T}{VS}^{T}\right)}_{\cdot i}}{s_{i\cdot }V^{T}Vs_{i\cdot }^{T}}{]}_{+},\\ v_{\cdot j} & \leftarrow & v_{\cdot j}+[\frac{{\left(X^{T}US\right)}_{\cdot j}-{\left({VS}^{T}U^{T}US\right)}_{\cdot j}}{s_{\cdot j}^{T}U^{T}Us_{\cdot j}}{]}_{+},\\ s_{ij} & \leftarrow & s_{ij}+[\frac{{\left(U^{T}XV\right)}_{ij}-{\left(U^{T}{USV}^{T}V\right)}_{ij}}{u_{\cdot i}^{T}u_{\cdot i}v_{\cdot j}^{T}v_{\cdot j}}{]}_{+}. \end{array}$$ (9)

Here, *u*_⋅*i*_ represents *i*-th column of **U**, and *u*_*i*⋅_ represents *i*-th row of **U**. Update rules for **U** and **V** successively applied to every column in **U** and **V**, where *s*_*ij*_ update is applied to each element in latent matrix **S**. Section D in S1 File shows full derivation of coordinate descent rules.

### Overview of optimization algorithms for non-negative matrix tri-factorization

Optimization methods considered in this paper use the same overall algorithmic approach shown in Algorithm 1. The main difference between these methods is the use of different update rules for latent matrices **U**, **S**, and **V**. The algorithm takes as input a data matrix **X**, and factorization rank parameters *k*_1_ and *k*_2_, which define the number of latent vectors for each dimension of the input matrix. Parameter *ϵ* defines the stopping criterion. First, the algorithm initializes latent matrices and fills them with random values. It then performs a series of iterations, during which it iteratively improves **U**, **V**, and **S** using appropriate equations. Multiplicative update rules method uses Eqs 2–4, alternating least squares method uses Eq 5, projected gradients method uses Eqs 6–8, and coordinate descent method uses Eq 9.

**Algorithm 1** Algorithm for non-negative matrix tri-factorization of **X** into latent matrices **U**, **S**, and **V**. MUR, multiplicative update rules; ALS, alternating least squares; PG, projected gradients; COD, coordinate descent.

**Input**: Data matrix $X\in R_{+}^{n\times m}$, Factorization ranks *k*_1_, *k*_2_ and optimization technique OPT.

1: Initialize $U^{n\times k_{1}}∼U\left(0,1\right)$

2: Initialize $V^{m\times k_{2}}∼U\left(0,1\right)$

3: Initialize $S^{k_{1}\times k_{2}}∼U\left(0,1\right)$

4: **repeat**

5:  **switch** (OPT)

6:  **case** MUR:

7:   Update **U**, **V**, **S** using Eqs 2, 3 and 4

8:  **case** ALS:

9:   Update **U**, **V**, **S** using Eq 5

10:  **case** PG:

11:   Update **U**, **V**, **S** using Eqs 6, 7 and 8

12:  **case** COD:

13:   Update **U**, **V**, **S** using Eq 9

14:  **end switch**

15: **until U**, **V** and **S** converge or maximum number of iterations is exceeded

16: **return U**, **V** and **S**

### Implementation

Methods are implemented in Python and available at https://github.com/acopar/fast-nmtf. The experiments were run on a dual Xeon E5-2660v4 server with combined number of 24 cores. Matrix operations were performed using NumPy package, accelerated with the Intel MKL library. Support for sparse matrix representation was implemented using SciPy library.

## Results

We empirically study the convergence of the algorithms on six datasets of varying size and density. We find that traditional multiplicative update rules method has the worst performance. In contrast, coordinate descent converges 5 to 24 times faster than multiplicative update rules (Table 2) and up to 16 times faster when comparing the runtime (Table 3). Multiplicative update rules method outperforms alternating least squares on dense datasets, whereas alternating least squares achieves most promising results on sparse datasets.

**Table 2:** Number of iterations needed by NMTF training algorithms to converge (Eq 11, *ε* = 10^−6^). Symbol ∞ denotes no convergence. MUR, multiplicative update rules; ALS, alternating least squares; PG, projected gradients; COD, coordinate descent. The MUR/COD column shows a speed-up of coordinate descent relative to multiplicative update rules, *i.e*., the number of iterations of MUR divided by the number of iterations of COD.

Dataset	Dataset type	MUR	ALS	PG	COD	MUR/COD
AlphaDigit	dense	3641	∞	1444	**332**	10.97
Coil20	dense	13598	∞	6348	**566**	24.03
STRING	sparse	1516	**67**	579	114	13.30
MovieLens	sparse	2165	319	1029	**154**	14.06
Mutations	sparse	1293	**86**	486	149	8.68
Newsgroups	sparse	432	**70**	148	86	5.02

**Table 3:** Runtime of NMTF training algorithms. Shown is time in seconds until convergence of each optimization method, averaged across ten independent runs of the method. Runs that did not converge are excluded from reporting. MUR, multiplicative update rules; ALS, alternating least squares; PG, projected gradients; COD, coordinate descent. The MUR/CUD column shows a speed-up of coordinate descent relative to multiplicative update rules, *i.e*., the runtime of MUR divided by the runtime of COD.

Dataset	MUR	ALS	PG	COD	MUR/COD
AlphaDigit	7.1	∞	4.5	**1.8**	4.0
Coil20	295.4	∞	170.8	**18.0**	16.4
STRING	236.0	**10.1**	92.5	19.8	11.9
MovieLens	839.6	106.6	349.6	**51.7**	16.2
Mutations	67.5	**4.5**	29.5	10.5	6.4
Newsgroups	39.4	**6.7**	15.9	11.4	3.5

### Experimental setup

We quantify convergence of an NMTF optimization algorithm by recording the number of algorithm iterations and the optimization runtime. We run each NMTF optimization algorithm until the relative difference of approximation error between two successive iterations is below a user-specified threshold. In particular, in iteration *i*, we calculate the value of objective function *D*_*i*_, which is defined as the squared Frobenius distance between input data matrix **X** and its approximation $\hat{X}=USV^{T}$ [52]:

$$\begin{array}{c} D_{i}=∥X-\hat{X}{∥}_{\text{Fro}}^{2}/{\left||X|\right|}_{\text{Fro}}^{2}=∥X-USV^{T}{∥}_{\text{Fro}}^{2}/{\left||X|\right|}_{\text{Fro}}^{2}, \end{array}$$ (10)

where **U**, **V**, **S** are the latent matrices returned in *i*-th iteration of the algorithm. Optimization is then terminated when the relative difference in objective function becomes sufficiently small [53]:

$$\begin{array}{c} |D_{i+1}-D_{i}|/D_{i}<\varepsilon , \end{array}$$ (11)

where *ε* = 10^−6^ is used in our experiments. Optimization method that needs fewer iterations to satisfy this stopping criterion is considered to represent a faster NMTF training algorithm under the assumption that the amount of computation required to execute one iteration is similar across different optimization methods. To avoid this assumption, we also the optimization runtime, *i.e*., the total amount of computation time needed to train the NMTF model until convergence.

**Fig 2:**
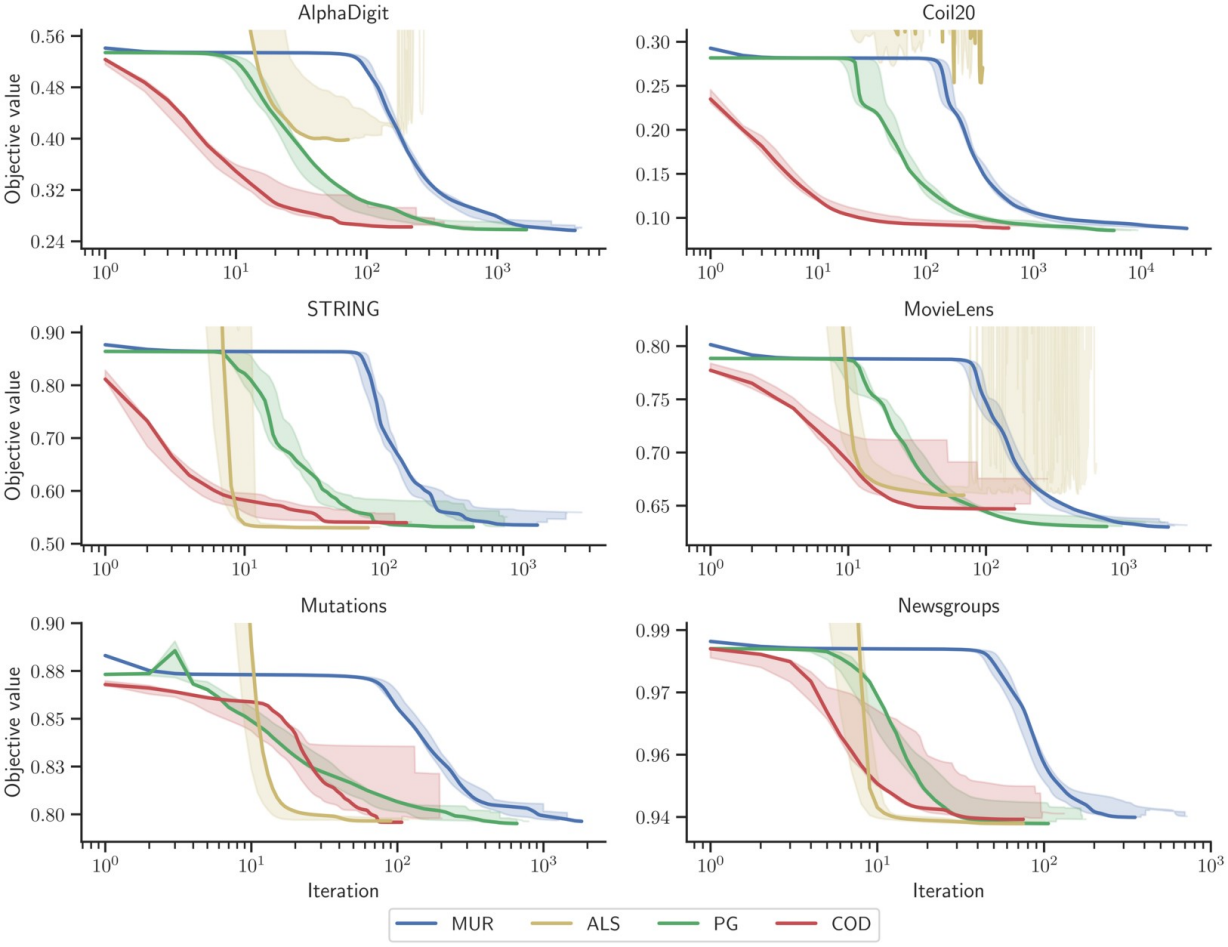
Optimization traces for six datasets and four NMTF optimization methods. Graphs show the value of NMTF cost (objective) function [4, 8, 14] at each iteration of the NMTF training algorithm. Shown is the optimization trace of the algorithm run with the smallest approximation error (solid lines). The highlighted area shows the span of the NMTF objective values across ten independent runs; each started from a different random initialization (see Experimental setup). MUR, multiplicative update rules; ALS, alternating least squares; PG, projected gradients; COD, coordinate descent.

We also qualitatively check convergence of NMTF training by tracing the value of the objective function (Fig 2) and we mark the training as diverging if the objective function oscillates or is at convergence point substantially higher than those of other optimization methods. In our experiments, we observed that alternating least squares method diverged on dense datasets. If the algorithm does not converge within a maximum number of iterations (*n*_STOP_ = 50,000), the optimization is terminated. If the algorithm does not reach the stopping criterion in *n*_STOP_ iteration, its results are excluded from reporting to avoid potential bias in results caused by selection of *n*_STOP_ parameter. Finally, in the case of multiplicative update rules methods, convergence in early iterations of training algorithm can be slow, which can accidentally trigger the stopping criterion. To address this issue, we additionally specify a minimum number of iterations (*n*_START_ = 100).

Non-negative matrix tri-factorization has two parameters, *k*_1_ and *k*_2_, that determine the size of latent matrices. We set these parameters to 20 in our analysis of convergence and we vary them (*k*_1_ = *k*_2_; *k*_*i*_ ∈ {10, 20, …, 100}) in order to study the impact of factorization rank on optimization runtime. We repeat all our experiments ten times and initialize latent matrices to values between 0 and 1 that are sampled uniformly at random [54].

### Convergence of NMTF optimization methods

Table 2 and Fig 2 show convergence of four NMTF optimization methods across six datasets. Table 2 reports the number of iterations needed by each optimization method to converge, averaged across ten independent runs of the method and omitting the runs in which the method does not converge. We see that alternating least squares and coordinate descent converge fastest and have a clear advantage over multiplicative update rules, a traditional NMTF optimization method. Additionally, our results suggest that coordinate descent might be most suitable for dense datasets, whereas alternating least squares method has poor convergence on dense datasets. Overall, considering optimization traces in Fig 2, coordinate descent converges fast and does not suffer from unstable training, which hampers alternative least squares. These results indicate that multiplicative update rules, which is the default NMTF optimization method in many applications, perform substantially worse than alternative optimization methods described in the present study.

Serizel *et al*. [47] show that two-factor NMF converges faster using a stochastic mini-batch approach, where the dataset is split into blocks and updates are in each iteration performed on each individual block. We have developed stochastic mini-batch versions of each of the four presented NMTF optimization techniques. Section G in S1 File and Fig C in S1 File show the convergence of mini-batch versions together with its non-batch counterparts. While the mini-batch variants do improve the convergence speed of multiplicative updates and projected gradient, they are highly unstable, and the resulting value of the objective function is worse compared to the non-mini-batch variants. Mini-batch variants of alternating least squares and coordinate descent did not converge.

### Analysis of matrix tri-factorization runtime

So far, we investigated convergence of NMTF optimization methods by studying the number of iterations needed by each method to converge. However, comparing methods solely based on the number of algorithm iterations is sufficient only if all methods perform an equal number of computations in each iteration. That is not true when training NMTF models (see Materials and methods). In particular, computational complexity of a single iteration of the algorithm varies substantially across optimization methods. It is thus essential to investigate and compare different methods by studying their optimization runtime.

Table 3 shows optimization runtime of four NMTF optimization methods. Results are qualitatively consistent with results in Table 2. Specifically, we find that coordinate descent excels on dense datasets, whereas alternating least squares method is the fastest method on sparse datasets.

### Impact of factorization rank on optimization runtime

Factorization rank is a crucial parameter of non-negative matrix tri-factorization (see Experimental setup) as it determines the size of latent matrices and, indirectly, the learning capacity of a factorized model. By increasing the number of latent vectors, *i.e*., increasing the values of *k*_1_ and *k*_2_, we can typically reduce the approximation error *D*_*i*_ (Eq 10); however larger factorization rank increases the runtime.

**Fig 3:**
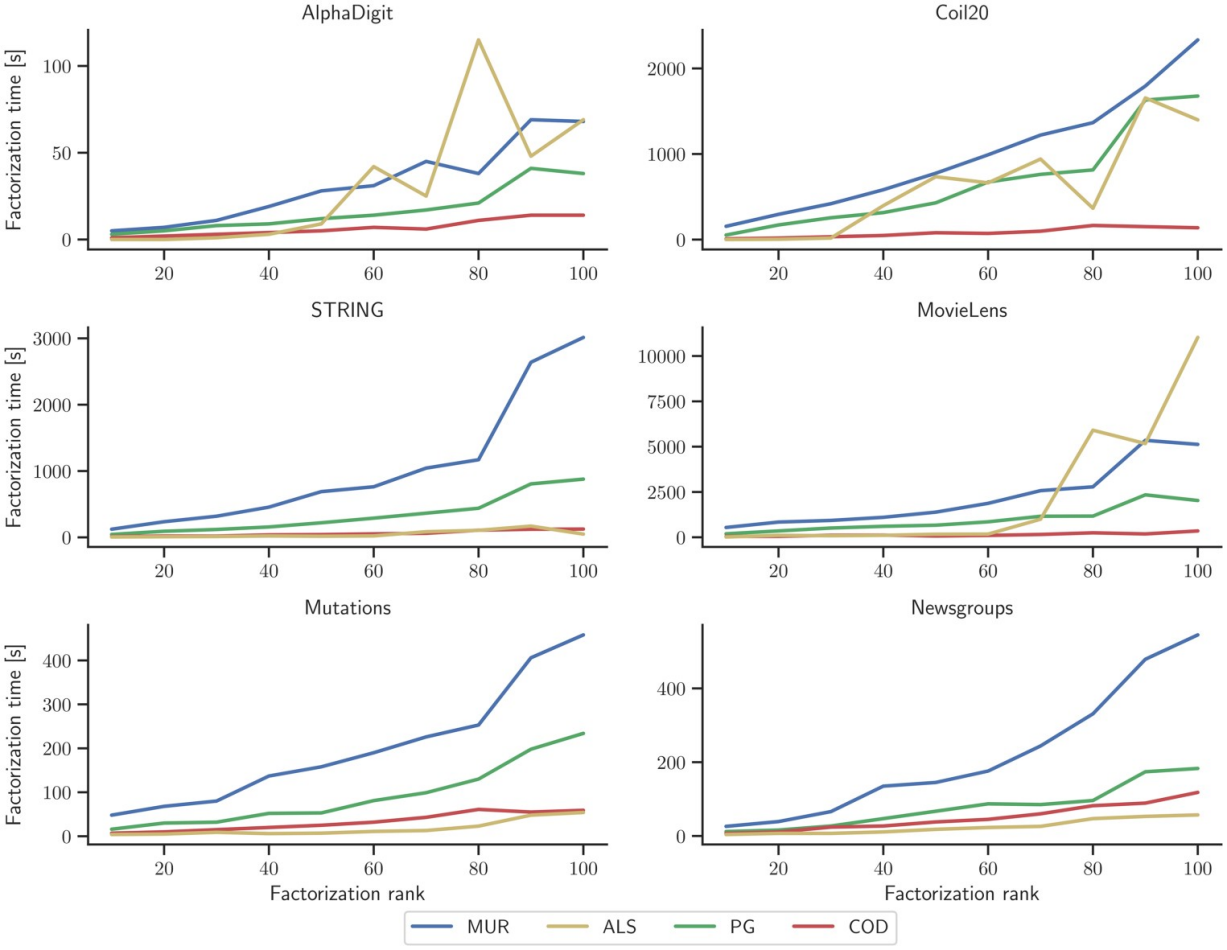
Impact of factorization rank on factorization time across six datasets and four NMTF optimization methods. The total runtime in seconds needed for convergence of the NMTF training algorithm is shown as a function of factorization rank (*k*_1_ = *k*_2_; *k*_*i*_ ∈ {10, 20, …, 100}), averaged across ten independent runs of the algorithm. Runs that did not converge are excluded from reporting. MUR, multiplicative update rules; ALS, alternating least squares; PG, projected gradients; COD, coordinate descent.

We studied how an increase in factorization rank affects the runtime of each of four NMTF optimization algorithms. Results in Fig 3 indicate that the runtime of multiplicative update rules and projected gradients increase much faster than the runtime for coordinate descent. Thus, we conclude that coordinate descent method might be the preferred optimization method in applications when large factorization rank is needed.

By increasing factorization rank, more latent vectors are added to the model. Larger factorization rank can lead to overfitting and with it to poorer generalization and can potentially affect performance on held-out data. To study the effects of factorization rank on objective value, we have varied the factorization rank in the range *k* ∈ {10, 20, …, 100} and assess the objective value on the held-out data transformed into the same latent space. Results (Section F in S1 File, Figs A and B in S1 File) are consistent with a similar experiment where the objective value was assessed on the training data (Fig 2). We also observe that coordinate descent and alternating least squares are more prone to fluctuations depending on the random initialization for larger factorization rank values.

## Discussion

Currently, multiplicative update rules represent a popular off-the-shelf optimization approach for non-negative matrix tri-factorization (NMTF) that is used in diverse applications, ranging from bioinformatics to natural language processing (*e.g*., [4–12]). We derived three new optimization methods for NMTF and demonstrated their convergence and scalability on six datasets of varying size and density. Importantly, we observe that coordinate descent, the newly derived method, converges fast and is stable on datasets of varying size and density. Our results suggest that coordinate descent might be a preferred off-the-shelf optimization method to train NMTF models on large datasets. These findings together with complete mathematical derivations (see S1 File) and a scalable public implementation of the algorithms (see section Implementation) represent primary contributions of this paper.

Coordinate descent offers a good compromise between factorization quality and the number of iterations of the algorithm needed for convergence. We find that coordinate descent is the fastest approach that often requires fewer than 100 iterations to converge, even on large datasets. Furthermore, the final value of the NMTF objective function attained by coordinate descent is comparable to that of multiplicative update rules. However, one drawback of coordinate descent is a higher computational cost per iteration, which can become an issue when factorizing data matrices at larger factorization ranks. Coordinate descent also exhibits higher sensitivity to initialization of the latent matrices, as indicated by the larger span of the objective function in Fig 2, especially in the case of small and sparse datasets.

The alternating least squares method performs well on sparse datasets but fails to converge to a high-quality solution on dense datasets. The method is thus sensitive to the properties of the dataset and, despite its performance on sparse data, we would advise using coordinate descent as a stable off-the-shelf NMTF optimization method. We note that the observed instabilities of alternating least squares and notable convergence issues are due to the heuristic enforcement of non-negativity in the learned latent matrices. In particular, as a final step in each iteration of the algorithm, alternating least squares method sets negative values in each latent matrix (**U**, **S**, and **V**) to zero values [19, 40]. The use of this heuristic generates non-negative latent matrices. However, the alternating least squares method cannot guarantee that the objective function value will decrease with each iteration of the algorithm, which can lead to instability of NMTF model training.

Our results suggest that multiplicative update rules method the most robust approach, as the method is not sensitive to initialization of latent matrices (Fig 2, see the width of the span of the NMTF objective function) and its final solution is at least as good as that of projected gradients or coordinate descent. However, multiplicative update rules method has the slowest convergence among the considered optimization methods. This finding is especially important as multiplicative update rules are currently favored NMTF optimization method. Another drawback of multiplicative update rules is a long start-up time; that is, the method appears to have reached a local stationary point during which the algorithm gives no improvements and returns latent matrices of low-quality if exited prematurely. A good alternative to multiplicative update rules are projected gradients. Similar to multiplicative update rules, projected gradients are robust and can learn a high-quality NMTF model, however, the methods needs an order of magnitude fewer iterations than multiplicative update rules and also has a shorter start-up time.

There are many interesting avenues of future work. For example, the use of heuristics could further improve performance of NMTF optimization methods [49]. Applying multiple updates to a particular latent matrix before moving on to updating the next latent matrix is a fruitful direction, as such approach could reduce the number of expensive matrix multiplications. Another idea is to use heuristics to determine the ordering of updates in the case of coordinate descent algorithm. In our experiments, coordinate descent used random initialization of latent matrices; however, pre-training by multiplicative update rules might further improve convergence.

Non-negative matrix tri-factorization is a core component of joint matrix factorization [8] that has been successfully used for fusion of heterogeneous data [55–57]. Such matrix factorization-based data integration can fuse many large datasets [10], however it can require substantial computational resources for inference. A speed-up of non-negative matrix tri-factorization by coordinate descent thus provides a fruitful research direction towards a computationally-effective data fusion and large-scale data integration.

## Conclusion

A traditional optimization approach to non-negative matrix tri-factorization uses multiplicative update rules. We described three alternative algorithms that train a non-negative matrix tri-factorization model based on alternating least squares, projected gradients, or coordinate descent. We conducted an empirical study comparing convergence and runtime of the training algorithms on six datasets. Our results show that the new approaches converge faster than multiplicative update rules and that coordinate descent achieves the best average performance.

## Supporting information

S1 File Detailed mathematical derivations for multiplicative updates, alternating least squares, projected gradient, coordinate descent and quasi-Newton optimization techniques. Experimental results of objective value on held-out data and convergence of stochastic mini-batch NMTF approach.
(PDF)
