# Supplementary material for: Fast optimization of non-negative matrix tri-factorization
Source: PLoS One. 2019 Jun 11;14(6):e0217994. doi: 10.1371/journal.pone.0217994 (PMC6559648; doi:10.1371/journal.pone.0217994)
Supplement: S1 File — Experimental results of objective value on held-out data and convergence of stochastic mini-batch NMTF approach. (PDF) [file pone.0217994.s001.pdf]

Fast Optimization of Non-Negative Matrix Tri-Factorization: Supporting Information

Andrej Čopar[1*], Blaž Zupan[1,2], and Marinka Zitnik[3,1]

[1]Faculty of Computer and Information Science, University of Ljubljana, Ljubljana, SI 1000, Slovenia
[2]Department of Molecular and Human Genetics, Baylor College of Medicine, Houston, TX 77030, USA
[3]Department of Computer Science, Stanford University, Stanford, CA 94305, USA
[*]andrej.copar@fri.uni-lj.si

OVERVIEW

An established method for non-negative matrix tri-factorization is based on multiplicative update rules. For completion, the derivations of these rules are summarized in Section A. Update rules for three alternative are derived here and represent one of the main contributions of this paper: Section B shows alternating least squares derivations, Section C projected gradient derivations, and Section D coordinate descent derivations. In Section E we show the equivalence between Quasi-Newton and ALS optimization technique for NMTF. Section F shows how the proposed approaches can be used to reconstruct previously unseen data into the same latent space. Section G shows results of mini-batch NMTF variants.

A Derivation of Multiplicative Update Rules

Multiplicative update rules for non-negative matrix tri-factorization (NMTF) and an orthogonal variant were introduced (Long et al., 2005, Ding et al., 2006). We focus on the non-orthogonal variant (Long et al., 2005) to make the approach comparable with alternative optimization techniques without added constraints. Both methods and all derivations in the following sections are based on the squared Frobenius norm objective function:

$$D_{Fro}(\mathbf{X}||\mathbf{USV}^T) = ||\mathbf{X}-\mathbf{USV}^T||^2_{Fro}., \tag{1}$$

where $\mathbf{X}$ presents the input data and $\mathbf{U}$, $\mathbf{S}$ and $\mathbf{V}$ are latent factors. Squared frobenius norm can be written as $||A||^2 = Tr(A^TA)$, therefore D_{Fro} equals to:

$$D_{Fro}(\mathbf{X}||\mathbf{USV}^T) = Tr(\mathbf{X}^T\mathbf{X} - 2\mathbf{X}^T\mathbf{USV}^T + \mathbf{VS}^T\mathbf{U}^T\mathbf{USV}^T). \tag{2}$$

Its partial derivatives with respect to each factor $\mathbf{U}$, $\mathbf{V}$, $\mathbf{S}$ are:

$$\frac{\partial F}{\partial \mathbf{U}} = -2\mathbf{XVS}^T + 2\mathbf{USV}^T\mathbf{VS}^T, \tag{3}$$

$$\frac{\partial F}{\partial \mathbf{V}} = -2\mathbf{X}^T\mathbf{US} + 2\mathbf{VS}^T\mathbf{U}^T\mathbf{US}, \tag{4}$$

$$\frac{\partial F}{\partial \mathbf{S}} = -2\mathbf{U}^T\mathbf{XV} + 2\mathbf{U}^T\mathbf{USV}^T\mathbf{V}. \tag{5}$$

Using the Karush-Kuhn Tucker (KKT) complementarity conditions, we can find a static point. The KKT condition for factor $\mathbf{U}$ is as follows:

$$\frac{\partial F}{\partial \mathbf{U}}_{ik} U_{ik} = 0.$$

This condition holds if $\frac{\partial F}{\partial \mathbf{U}}$ reached a static point (the derivative is zero):

$$(\mathbf{USV}^T\mathbf{VS}^T - \mathbf{XVS}^T)_{ik}U_{ik} = 0, \tag{6}$$

$$U_{ik} = U_{ik}\frac{(\mathbf{XVS}^T)_{ik}}{(\mathbf{USV}^T\mathbf{VS}^T)_{ik}}.$$

Similarly, we take the KKT complementarity condition for $\mathbf{V}$ and derive the second update rule:

$$\begin{aligned}
\frac{\partial F}{\partial \mathbf{V}_{ik}} V_{ik} &= 0, \\
(\mathbf{V}\mathbf{S}^T\mathbf{U}^T\mathbf{U}\mathbf{S} - \mathbf{X}^T\mathbf{U}\mathbf{S})_{ik} V_{ik} &= 0, \\
V_{ik} &= V_{ik} \frac{(\mathbf{X}^T\mathbf{U}\mathbf{S})_{ik}}{(\mathbf{V}\mathbf{S}^T\mathbf{U}^T\mathbf{U}\mathbf{S})_{ik}}.
\end{aligned} \tag{7}$$

The third update rule is computed with respect to factor $\mathbf{S}$:

$$\begin{aligned}
\frac{\partial F}{\partial \mathbf{S}_{ik}} S_{ik} &= 0, \\
(\mathbf{U}^T\mathbf{U}\mathbf{S}\mathbf{V}^T\mathbf{V} - \mathbf{U}^T\mathbf{X}\mathbf{V}) S_{ik} &= 0, \\
S_{ik} &= S_{ik} \frac{\mathbf{U}^T\mathbf{X}\mathbf{V}}{\mathbf{U}^T\mathbf{U}\mathbf{S}\mathbf{V}^T\mathbf{V}}.
\end{aligned} \tag{8}$$

These rules can be expressed in matrix form, where $\odot$ denotes Hadamard product and symbol $\oslash$ denotes Hadamard division:

$$\mathbf{U} \leftarrow \mathbf{U} \odot (\mathbf{X}\mathbf{V}\mathbf{S}^T \oslash \mathbf{U}\mathbf{S}\mathbf{V}^T\mathbf{V}\mathbf{S}^T), \tag{9}$$
$$\mathbf{V} \leftarrow \mathbf{V} \odot (\mathbf{X}^T\mathbf{U}\mathbf{S} \oslash \mathbf{V}\mathbf{S}^T\mathbf{U}^T\mathbf{U}\mathbf{S}), \tag{10}$$
$$\mathbf{S} \leftarrow \mathbf{S} \odot (\mathbf{U}^T\mathbf{X}\mathbf{V} \oslash \mathbf{U}^T\mathbf{U}\mathbf{S}\mathbf{V}^T\mathbf{V}). \tag{11}$$

B Derivation of Alternating Least Squares

Let us calculate the gradient of function Eq. (1) with respect to each factor matrix $\mathbf{U}$, $\mathbf{V}$ and $\mathbf{S}$. We follow the derivations in Eq. (3), (4) and (5) and simplify the equations:

$$\begin{aligned}
\frac{\partial F}{\partial \mathbf{U}} &= \mathbf{X}\mathbf{V}\mathbf{S}^T - \mathbf{U}\mathbf{S}\mathbf{V}^T\mathbf{V}\mathbf{S}^T, \\
\frac{\partial F}{\partial \mathbf{V}} &= \mathbf{X}^T\mathbf{U}\mathbf{S} - \mathbf{V}\mathbf{S}^T\mathbf{U}^T\mathbf{U}\mathbf{S}, \\
\frac{\partial F}{\partial \mathbf{S}} &= \mathbf{U}^T\mathbf{X}\mathbf{V} - \mathbf{U}^T\mathbf{U}\mathbf{S}\mathbf{V}^T\mathbf{V}.
\end{aligned} \tag{12}$$

We equate the gradient for each factor to zero $\frac{\partial F}{\partial \mathbf{U}} = 0$. After this step, we calculate the inverse to get the update rule for $\mathbf{U}$:

$$\begin{aligned}
\mathbf{U}\mathbf{S}\mathbf{V}^T\mathbf{V}\mathbf{S}^T &= \mathbf{X}\mathbf{V}\mathbf{S}^T, \\
\mathbf{U} &= \mathbf{X}\mathbf{V}\mathbf{S}^T(\mathbf{S}\mathbf{V}^T\mathbf{V}\mathbf{S}^T)^{-1}.
\end{aligned} \tag{13}$$

This step can introduce negative values, so we must force non-negativity by assigning all negative elements with zero:

$$\mathbf{U} = \left[\mathbf{X}\mathbf{V}\mathbf{S}^T(\mathbf{S}\mathbf{V}^T\mathbf{V}\mathbf{S}^T)^{-1}\right]_+.$$

Following the derivative for $\mathbf{V}$ from Eq. (4), we can similarly formulate update rule for $\mathbf{V}$. We equate the gradient with zero $\frac{\partial F}{\partial \mathbf{V}} = 0$, calculate the inverse and force non-negativity:

$$\begin{aligned}
\mathbf{S}^T\mathbf{U}^T\mathbf{U}\mathbf{S}\mathbf{V}^T &= \mathbf{S}^T\mathbf{U}^T\mathbf{X}, \\
\mathbf{V}^T &= (\mathbf{S}^T\mathbf{U}^T\mathbf{U}\mathbf{S})^{-1}\mathbf{S}^T\mathbf{U}^T\mathbf{X}, \\
\mathbf{V} &= \mathbf{X}^T\mathbf{U}\mathbf{S}(\mathbf{S}^T\mathbf{U}^T\mathbf{U}\mathbf{S})^{-1}, \\
\mathbf{V} &= \left[\mathbf{X}^T\mathbf{U}\mathbf{S}(\mathbf{S}^T\mathbf{U}^T\mathbf{U}\mathbf{S})^{-1}\right]_+.
\end{aligned} \tag{14}$$

Derivative for $\mathbf{S}$ is shown in equation (5). We set the gradient $\frac{\partial F}{\partial \mathbf{S}}$ to zero and force non-negativity constraints:

$$\begin{aligned}
\mathbf{U}^T\mathbf{U}\mathbf{S}\mathbf{V}^T\mathbf{V} &= \mathbf{U}^T\mathbf{X}\mathbf{V}, \\
\mathbf{S} &= (\mathbf{U}^T\mathbf{U})^{-1}(\mathbf{U}^T\mathbf{X}\mathbf{V})(\mathbf{V}^T\mathbf{V})^{-1}, \\
\mathbf{S} &= \left[(\mathbf{U}^T\mathbf{U})^{-1}(\mathbf{U}^T\mathbf{X}\mathbf{V})(\mathbf{V}^T\mathbf{V})^{-1}\right]_+.
\end{aligned} \tag{15}$$

C Derivation of Projected Gradients

Projected gradient methods are based on the general gradient descent scheme, where we take the variable X and create a step towards the descent direction P scaled with the learning rate η:

$$X \leftarrow X - \eta P. \tag{16}$$

We derive projected gradient algorithm for NMTF, for the squared Frobenius norm objective function (1). We add projection to the non-negative values $[\,]_+$ to enforce non-negativity of the factors in case in crosses into negative values.
Projected gradient methods follow this form:

$$\begin{aligned} \mathbf{U} &\leftarrow [\mathbf{U} - \eta_u \mathbf{P}_U]_+ , \\ \mathbf{S} &\leftarrow [\mathbf{S} - \eta_s \mathbf{P}_S]_+ , \\ \mathbf{V} &\leftarrow [\mathbf{V} - \eta_v \mathbf{P}_V]_+ . \end{aligned} \tag{17}$$

where $\mathbf{P}_U$, $\mathbf{P}_V$ and $\mathbf{P}_S$ are descent directions, η_u, η_v and η_s are learning rates. Descent directions are defined with the following form:

$$\begin{aligned} \mathbf{P}_U &= D_U \odot \frac{\partial F}{\partial \mathbf{U}}, \\ \mathbf{P}_V &= D_V \odot \frac{\partial F}{\partial \mathbf{V}}, \\ \mathbf{P}_S &= D_S \odot \frac{\partial F}{\partial \mathbf{S}}. \end{aligned} \tag{18}$$

The scaling factors $\mathbf{D}_U$, $\mathbf{D}_V$ and $\mathbf{D}_S$ are set the following way:

$$\begin{aligned} \mathbf{D}_U &= \mathbf{U} \oslash (\mathbf{U}\mathbf{S}\mathbf{V}^T\mathbf{V}\mathbf{S}^T), \\ \mathbf{D}_V &= \mathbf{V} \oslash (\mathbf{V}\mathbf{S}^T\mathbf{U}^T\mathbf{U}\mathbf{S}), \\ \mathbf{D}_S &= \mathbf{S} \oslash (\mathbf{U}^T\mathbf{U}\mathbf{S}\mathbf{V}^T\mathbf{V}). \end{aligned} \tag{19}$$

The scaling factor was chosen based on a study which compares convergence analysis of four different scaling factors (Merritt, 2005). The choice of scaling factor is inspired by existing projected gradient algorithm for non-negative matrix factorization (Lin, 2007). The learning rate or step size parameter is dynamically chosen using the same form as a related study for classical non-negative matrix factorization (Lin, 2007):

$$\eta_u = Tr((\mathbf{S}\mathbf{V}^T\mathbf{V})(\mathbf{S}^T\mathbf{P}_U^T\mathbf{P}_U)).$$

We calculate the descent direction for each factor matrix $\mathbf{U}$ using the descent direction for $\mathbf{U}$ and the partial derivative (3):

$$\begin{aligned} \mathbf{P}_U &= (\mathbf{U} \oslash \mathbf{U}\mathbf{S}\mathbf{V}^T\mathbf{V}\mathbf{S}^T) \odot (\mathbf{X} - \mathbf{U}\mathbf{S}\mathbf{V}^T)\mathbf{V}\mathbf{S}^T, \\ \mathbf{P}_U &= \mathbf{U} - \mathbf{U} \oslash \mathbf{U}\mathbf{S}\mathbf{V}^T\mathbf{V}\mathbf{S}^T \odot \mathbf{X}\mathbf{V}\mathbf{S}^T. \end{aligned} \tag{20}$$

We define learning rate for $\mathbf{V}$ as follows:

$$\eta_v = Tr((\mathbf{S}\mathbf{P}_V^T\mathbf{P}_V)(\mathbf{S}\mathbf{U}^T\mathbf{U})).$$

We calculate the descent direction for $\mathbf{V}$ by inserting the D_S and derivative in Eq. (4) into the Eq. (19):

$$\begin{aligned} \mathbf{P}_V &= (\mathbf{V} \oslash \mathbf{V}\mathbf{S}^T\mathbf{U}^T\mathbf{U}\mathbf{S}) \odot (\mathbf{X} - \mathbf{U}\mathbf{S}\mathbf{V}^T)^T\mathbf{U}\mathbf{S}, \\ \mathbf{P}_V &= \mathbf{V} - \mathbf{V} \oslash \mathbf{V}\mathbf{S}^T\mathbf{U}^T\mathbf{U}\mathbf{S} \odot \mathbf{X}^T\mathbf{U}\mathbf{S}. \end{aligned} \tag{21}$$

We define learning rate for $\mathbf{S}$ as follows:

$$\eta_s = Tr((\mathbf{U}^T\mathbf{U}\mathbf{P}_S)(\mathbf{V}^T\mathbf{V}\mathbf{P}_S^T)).$$

The descent direction for $\mathbf{S}$ is computed by inserting the D_S and derivative in Eq. (5) into the Eq. (19):

$$\begin{aligned} \mathbf{P}_S &= (\mathbf{S} \oslash \mathbf{U}^T\mathbf{U}\mathbf{S}\mathbf{V}^T\mathbf{V}) \odot \mathbf{U}^T(\mathbf{X} - \mathbf{U}\mathbf{S}\mathbf{V}^T)\mathbf{V}, \\ \mathbf{P}_S &= \mathbf{S} - \mathbf{S} \oslash \mathbf{U}^T\mathbf{U}\mathbf{S}\mathbf{V}^T\mathbf{V} \odot \mathbf{U}^T\mathbf{X}\mathbf{V}. \end{aligned} \tag{22}$$

We insert the descent directions and learning rates in Eq. (18). The resulting update rules for **U** are:

$$\begin{aligned}
\mathbf{P_u} &= \mathbf{U} - \mathbf{U} \oslash (\mathbf{USV}^T\mathbf{VS}^T) \odot (\mathbf{XVS}^T), \\
\eta_u &= \frac{\sum(\mathbf{P_u} \odot (\mathbf{USV}^T\mathbf{VS}^T - \mathbf{XVS}^T))}{Tr((\mathbf{SV}^T\mathbf{V})(\mathbf{S}^T\mathbf{P_u}^T\mathbf{P_u}))}, \\
\mathbf{U} &\leftarrow [\mathbf{U} - \eta_u \mathbf{P_u}]_+ .
\end{aligned} \tag{23}$$

The following update rules define the prodecude for updating factor **V**:

$$\begin{aligned}
\mathbf{P_v} &= \mathbf{V} - \mathbf{V} \oslash (\mathbf{VS}^T\mathbf{U}^T\mathbf{US}) \odot (\mathbf{X}^T\mathbf{US}), \\
\eta_v &= \frac{\sum(\mathbf{P_v} \odot (\mathbf{VS}^T\mathbf{U}^T\mathbf{US} - \mathbf{X}^T\mathbf{US}))}{Tr((\mathbf{SP_v}^T\mathbf{P_v})(\mathbf{S}^T\mathbf{U}^T\mathbf{U}))}, \\
\mathbf{V} &\leftarrow [\mathbf{V} - \eta_v \mathbf{P_v}]_+ .
\end{aligned} \tag{24}$$

Resulting update rules for factor **S** are:

$$\begin{aligned}
\mathbf{P_s} &= \mathbf{S} - \mathbf{S} \oslash (\mathbf{U}^T\mathbf{USV}^T\mathbf{V}) \odot (\mathbf{U}^T\mathbf{XV}), \\
\eta_s &= \frac{\sum(\mathbf{P_s} \odot (\mathbf{U}^T\mathbf{USV}^T\mathbf{V} - \mathbf{U}^T\mathbf{XV}))}{Tr((\mathbf{U}^T\mathbf{UP_s})(\mathbf{V}^T\mathbf{VP_s}^T))}, \\
\mathbf{S} &\leftarrow [\mathbf{S} - \eta_s \mathbf{P_s}]_+ .
\end{aligned} \tag{25}$$

D Derivation of Coordinate Descent

First, we define the following residual matrices:

$$\mathbf{X}^i = \mathbf{X} - \mathbf{USV}^T + u_{\cdot i}(\mathbf{SV}^T)_{\cdot i}, \tag{26}$$

$$\mathbf{X}^j = \mathbf{X} - \mathbf{USV}^T + (\mathbf{US})_{\cdot j} v_{\cdot j}^T, \tag{27}$$

$$\mathbf{X}^{(i,j)} = \mathbf{X} - \mathbf{USV}^T + (u_{\cdot i} s_{ij} v_{\cdot j}^T). \tag{28}$$

The idea is to iterate over this set of subproblems for $i \in \{1 \dots k_1\}$ and $j \in \{1 \dots k_2\}$:

$$\begin{aligned}
F_U^{(i)}(u_i) &= ||\mathbf{X}^i - u_i(\mathbf{SV}^T)_i||^2 = ||\mathbf{X}^i - u_i s_{i\cdot}\mathbf{V}^T||^2, \\
F_V^{(j)}(v_j) &= ||\mathbf{X}^j - (\mathbf{US})_j v_{\cdot j}^T||^2 = ||\mathbf{X}^j - \mathbf{U}s_{\cdot j} v_{\cdot j}^T||^2, \\
F_S^{(ij)}(s_{ij}) &= ||\mathbf{X}^{(i,j)} - u_{\cdot i} s_{ij} v_{\cdot j}^T||^2.
\end{aligned} \tag{29}$$

We use $||A||_2 = Tr(A^TA)$ and $(AB)_{\cdot i} = Ab_{\cdot i}$, where $b_{\cdot i}$ is i-th column of B. First, we derive the update rule for $F_U^{(i)}(u_i)$:

$$F_U^{(i)}(u_i) = Tr(\mathbf{X}^{(i)^T}\mathbf{X}^{(i)} - 2\mathbf{X}^{(i)^T} u_{\cdot i} s_{i\cdot}\mathbf{V}^T + \mathbf{V}s_{i\cdot}^T u_{\cdot i}^T u_{\cdot i} s_{i\cdot}\mathbf{V}^T), \tag{30}$$

$$\frac{\partial F}{\partial u_i} = -2\mathbf{X}^{(i)}\mathbf{V}s_{i\cdot}^T + 2u_{\cdot i}s_{i\cdot}\mathbf{V}^T\mathbf{V}s_{i\cdot}^T .$$

Then, we equate the derivative $\frac{\partial F}{\partial u_i}$ to zero:

$$\begin{aligned}
\mathbf{X}^i\mathbf{V}s_{i\cdot}^T &= u_{\cdot i}s_{i\cdot}\mathbf{V}^T\mathbf{V}s_{i\cdot}^T, \\
u_{\cdot i} &= \frac{\mathbf{X}^i\mathbf{V}s_{i\cdot}^T}{s_{i\cdot}\mathbf{V}^T\mathbf{V}s_{i\cdot}^T}.
\end{aligned} \tag{31}$$

Update for the $F_V^{(j)}(v_j)$ subproblem:

$$F_V^{(j)}(v_j) = Tr(\mathbf{X}^{(j)^T}\mathbf{X}^{(j)} - 2\mathbf{X}^{(j)^T}\mathbf{U}s_j v_{\cdot j}^T + v_{\cdot j}s_{\cdot j}^T\mathbf{U}^T\mathbf{U}s_j v_{\cdot j}^T), \tag{32}$$

$$\frac{\partial F}{\partial v_j} = -2\mathbf{X}^{(j)^T}\mathbf{U}s_j + 2v_{\cdot j}s_j^T\mathbf{U}^T\mathbf{U}s_j .$$

We equate the derivative $\frac{\partial F}{\partial v_j}$ to zero:

$$\begin{aligned} \mathbf{X}^{j^T}\mathbf{U}s_j &= v_{\cdot j}s_j^T\mathbf{U}^T\mathbf{U}s_{\cdot j}, \\ v_{\cdot j} &= \frac{\mathbf{X}^{j^T}\mathbf{U}s_{\cdot j}}{s_{\cdot j}^T\mathbf{U}^T\mathbf{U}s_{\cdot j}}. \end{aligned} \tag{33}$$

Finally, we find the static point for the $F_S^{(ij)}(s_{ij})$ subproblem:

$$\begin{aligned} F_S^{(ij)}(s_{ij}) &= Tr(\mathbf{X}^{(i,j)^T}\mathbf{X}^{(i,j)} - 2\mathbf{X}^{(i,j)^T}u_{\cdot i}s_{ij}v_{\cdot j}^T + v_{\cdot j}s_{ij}^Tu_{\cdot i}^Tu_{\cdot i}s_{ij}v_{\cdot j}^T), \\ \frac{\partial F}{\partial s_{ij}} &= -2u_{\cdot i}^T\mathbf{X}^{(i,j)}v_{\cdot j} + 2u_{\cdot i}^Tu_{\cdot i}s_{ij}v_{\cdot j}^Tv_{\cdot j}. \end{aligned} \tag{34}$$

We equate the derivative $\frac{\partial F}{\partial s_{ij}}$ to zero:

$$\begin{aligned} u_{\cdot i}^T\mathbf{X}^{(i,j)}v_{\cdot j} &= u_{\cdot i}^Tu_{\cdot i}s_{ij}v_{\cdot j}^Tv_{\cdot j}, \\ s_{ij} &= \frac{u_{\cdot i}^T\mathbf{X}^{(i,j)}v_{\cdot j}}{u_{\cdot i}^Tu_{\cdot i}v_{\cdot j}^Tv_{\cdot j}}. \end{aligned} \tag{35}$$

For efficient implementation, we replace residual matrices $\mathbf{X}^i$, $\mathbf{X}^j$ and $\mathbf{X}^{i,j}$ with definitions in equations (26), (27) and (28). We also enforce non-negativity with projection to non-negative values. The resulting update rules are:

$$u_{\cdot i} \leftarrow u_{\cdot i} + \left[\frac{(\mathbf{XVS}^T)_{\cdot i} - (\mathbf{USV}^T\mathbf{VS}^T)_{\cdot i}}{s_{i\cdot}\mathbf{V}^T\mathbf{V}s_{i\cdot}^T}\right]_+, \tag{36}$$

$$v_{\cdot j} \leftarrow v_{\cdot j} + \left[\frac{(\mathbf{X}^T\mathbf{US})_{\cdot j} - (\mathbf{VS}^T\mathbf{U}^T\mathbf{US})_{\cdot j}}{s_{\cdot j}^T\mathbf{U}^T\mathbf{U}s_{\cdot j}}\right]_+, \tag{37}$$

$$s_{ij} \leftarrow s_{ij} + \left[\frac{(\mathbf{U}^T\mathbf{XV})_{ij} - (\mathbf{U}^T\mathbf{USV}^T\mathbf{V})_{ij}}{u_{\cdot i}^Tu_{\cdot i}v_{\cdot j}^Tv_{\cdot j}}\right]_+. \tag{38}$$

These rules appear in the additive form, where the step size η_u is implicitly defined within the update rules:

$$u_{\cdot i} = u_{\cdot i} + \left[\eta_u\left(\frac{\partial F}{\partial U}\right)_{\cdot i}\right]_+. \tag{39}$$

From the equation (36) and (3) we can observe the step size:

$$\eta_u = 1/(s_i\mathbf{V}^T\mathbf{V}s_i^T). \tag{40}$$

Similarly, the step sizes for $v_{\cdot j}$ and s_{ij} can be observed:

$$\eta_v = 1/s_{\cdot j}^T\mathbf{U}^T\mathbf{U}s_{\cdot j}, \tag{41}$$

$$\eta_s = 1/u_{\cdot i}^Tu_{\cdot i}v_{\cdot j}^Tv_{\cdot j}. \tag{42}$$

We observed that such step size is effective in preventing the values to cross into negative space. The loss of information associated with projection into non-negative space is reduced, which keeps convergence stable throughout the factorization procedure.

E Derivation of Quasi-Newton update rules

In this section we show the equivalence of quasi-Newton update rules and ALS. The quasi-Newton update rules are formulated as follows, where $\mathbf{H}_U$, $\mathbf{H}_V$, and $\mathbf{H}_S$ are Hessians of each factors:

$$\mathrm{vec}(\mathbf{U}) \leftarrow \left[\mathrm{vec}(\mathbf{U}) - \mathbf{H}_U^{-1}\,\mathrm{vec}\left(\frac{\partial F}{\partial \mathbf{U}}\right)\right]_+, \tag{43}$$

$$\mathrm{vec}(\mathbf{V}) \leftarrow \left[\mathrm{vec}(\mathbf{V}) - \mathbf{H}_V^{-1}\,\mathrm{vec}\left(\frac{\partial F}{\partial \mathbf{V}}\right)\right]_+, \tag{44}$$

$$\mathrm{vec}(\mathbf{S}) \leftarrow \left[\mathrm{vec}(\mathbf{S}) - \mathbf{H}_S^{-1}\,\mathrm{vec}\left(\frac{\partial F}{\partial \mathbf{S}}\right)\right]_+. \tag{45}$$

Let us calculate the gradient and the Hessian of function Eq. (1) with respect to $\mathbf{U}$:

$$\begin{aligned} \frac{\partial F}{\partial \mathbf{U}} &= \mathbf{XVS}^T - \mathbf{USV}^T\mathbf{VS}^T, \quad (46) \\ \mathbf{H}_U &= \mathbf{SV}^T\mathbf{VS}^T \otimes \mathbf{I}. \quad (47) \end{aligned}$$

The Kronecker product is shown with $\otimes$, the vec-operator is shown with vec, and $\mathbf{I}$ is identity matrix. By placing the gradient and the Hessian into the equation (43), we get:

$$\text{vec}(\mathbf{U}) = \left[\text{vec}(\mathbf{U}) - (\mathbf{SV}^T\mathbf{VS}^T \otimes \mathbf{I})^{-1}\,\text{vec}(\mathbf{USV}^T\mathbf{VS}^T - \mathbf{XVS}^T)\right]_+, \quad (48)$$

which by converting from vec-operator to matrix form, using the rule $\text{vec}(ABC) = (C^T \otimes A)\,\text{vec}(B)$ simplifies to:

$$\mathbf{U} = \left[\mathbf{U} - (\mathbf{USV}^T\mathbf{VS}^T - \mathbf{XVS}^T)(\mathbf{SV}^T\mathbf{VS}^T)^{-1}\right]_+. \quad (49)$$

Further, we derive the gradient and the Hessian matrix of the factor $\mathbf{V}$:

$$\begin{aligned} \frac{\partial F}{\partial \mathbf{V}} &= \mathbf{X}^T\mathbf{US} - \mathbf{VS}^T\mathbf{U}^T\mathbf{US}, \quad (50) \\ \mathbf{H}_V &= \mathbf{S}^T\mathbf{U}^T\mathbf{US} \otimes \mathbf{I}. \quad (51) \end{aligned}$$

We place the gradient and Hessian into equation (44) and convert to matrix form:

$$\mathbf{V} = \left[\mathbf{V} - (\mathbf{VS}^T\mathbf{U}^T\mathbf{US} - \mathbf{X}^T\mathbf{US})(\mathbf{S}^T\mathbf{U}^T\mathbf{US})^{-1}\right]_+. \quad (52)$$

The gradient and the Hessian of the $\mathbf{S}$ matrix are:

$$\begin{aligned} \frac{\partial F}{\partial \mathbf{S}} &= \mathbf{U}^T\mathbf{XV} - \mathbf{U}^T\mathbf{USV}^T\mathbf{V}, \quad (53) \\ \mathbf{H}_S &= \mathbf{V}^T\mathbf{V} \otimes \mathbf{U}^T\mathbf{U}. \quad (54) \end{aligned}$$

We place the gradient and the Hessian into the equation (45).

$$\text{vec}(\mathbf{S}) = \left[\text{vec}(\mathbf{S}) - (\mathbf{V}^T\mathbf{V} \otimes \mathbf{U}^T\mathbf{U})^{-1}\,\text{vec}(\mathbf{USV}^T\mathbf{VS}^T - \mathbf{XVS}^T)\right]_+, \quad (55)$$

We use the following rule to convert from vector to the matrix form: $\text{vec}(ABC) = (C^T \otimes A)\,\text{vec}(B)$, where $A = (\mathbf{U}^T\mathbf{U})^{-1}$, $C = (\mathbf{V}^T\mathbf{V})^{-1}$, and $B = \frac{\partial F}{\partial \mathbf{S}}$. Note that $(\mathbf{V}^T\mathbf{V})^T = \mathbf{V}^T\mathbf{V}$ since $\mathbf{V}^T\mathbf{V}$ is symmetric.

$$\mathbf{S} = \left[\mathbf{S} - (\mathbf{U}^T\mathbf{U})^{-1}(\mathbf{U}^T\mathbf{USV}^T\mathbf{V} - \mathbf{U}^T\mathbf{XV})(\mathbf{V}^T\mathbf{V})^{-1}\right]_+. \quad (56)$$

Let us simplify the equations (49), (52) and (56), using the distributive rule $(A+B)C = AC + BC$:

$$\begin{aligned} \mathbf{U} &= \left[\mathbf{U} - \mathbf{U} + \mathbf{XVS}^T(\mathbf{SV}^T\mathbf{VS}^T)^{-1}\right]_+, \\ \mathbf{V} &= \left[\mathbf{V} - \mathbf{V} + \mathbf{X}^T\mathbf{US}(\mathbf{S}^T\mathbf{U}^T\mathbf{US})^{-1}\right]_+, \quad (57) \\ \mathbf{S} &= \left[\mathbf{S} - \mathbf{S} + (\mathbf{U}^T\mathbf{U})^{-1}(\mathbf{U}^T\mathbf{XV})(\mathbf{V}^T\mathbf{V})^{-1}\right]_+. \end{aligned}$$

These rules are equivalent to the ALS update rules. Note that similar was already shown for NMF by Cichocki.[1]

F Reconstruction on testing data

In this section we study reconstruction error of different optimization approaches on held-out data. We split the data $\mathbf{X}$ row-wise into two parts: $\mathbf{X}_{train}$ is composed of the first 80 percent and $\mathbf{X}_{new}$ composed of the remaining 20 percent of the data. Then, we run the factorization on $\mathbf{X}_{train}$, resulting in $\mathbf{U}_{train}$, $\mathbf{S}$ and $\mathbf{V}$. We transform the remaining data $\mathbf{X}_{new}$ into the latent space defined by $\mathbf{S}$ and $\mathbf{V}$, such that the following objective function is minimized:

$$D_{\text{Fro}}(\mathbf{X}_{new}||\mathbf{U}_{new}\mathbf{SV}^T) = ||\mathbf{X}_{new} - \mathbf{U}_{new}\mathbf{SV}^T||^2_{\text{Fro}}. \quad (58)$$

We keep the $\mathbf{S}$ and $\mathbf{V}$ factors from the training step and initialize $\mathbf{U}_{new}$ with random values. We iteratively apply updates on $\mathbf{U}_{new}$, while $\mathbf{S}$ and $\mathbf{V}$ remain unchanged. Figure A shows the objective value of four optimization techniques according to Eq. 58 at factorization rank $k_1, k_2 = 20$. Each experiment is repeated ten times until the convergence criterion with parameter $\varepsilon = 6$ is reached.

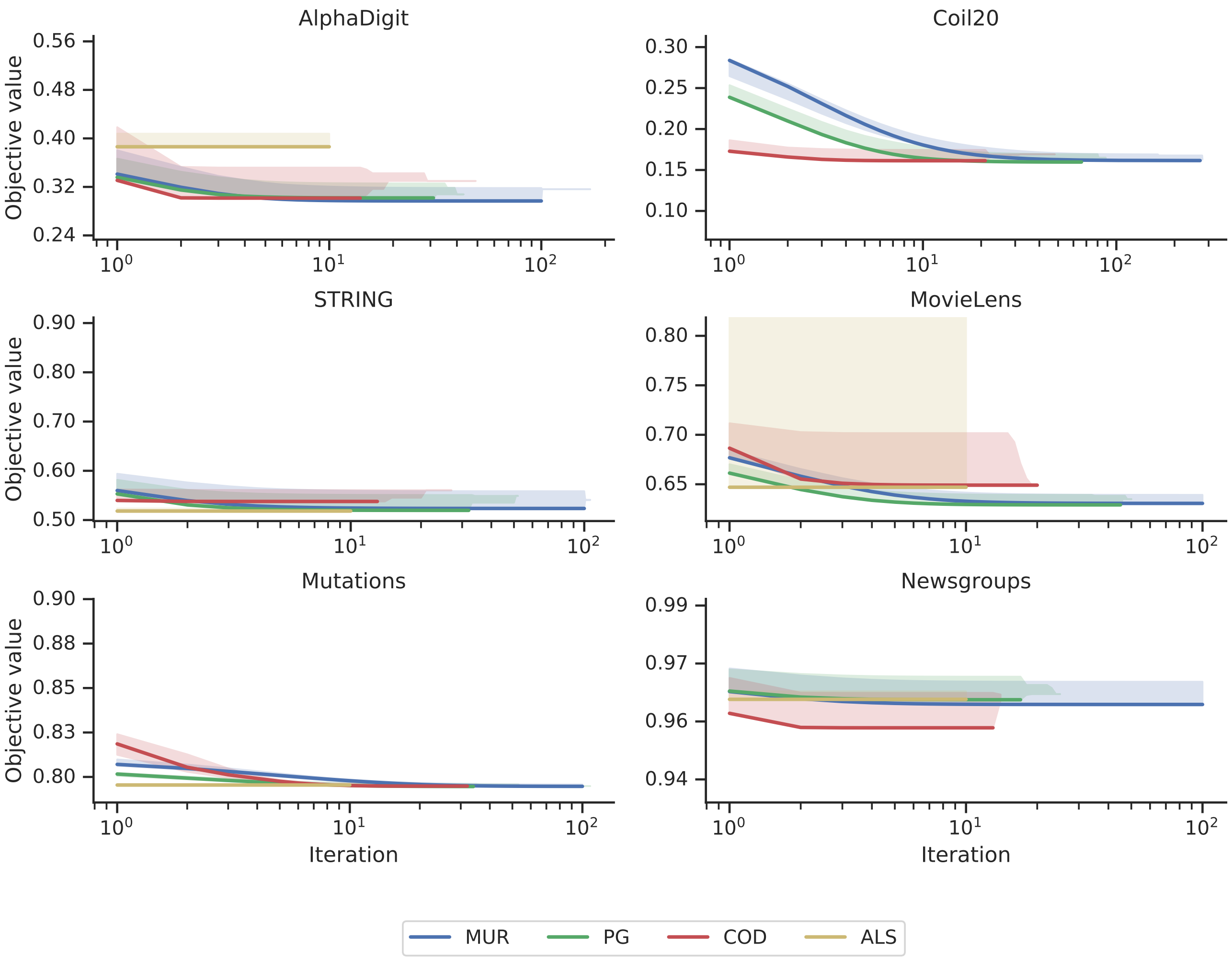

Figure A. Convergence of matrix factorization procedure on held-out (test) data of the factorization system that was pre-trained on the held-in (training) data. The experiments were repeated ten times, each time with different random sampling. Solid lines show the convergence of the run with the best-end objective value at convergence point, and the highlighted area shows the span of objective values. MUR, multiplicative update rules; ALS, alternating least squares; PG, projected gradients; COD, coordinate descent.

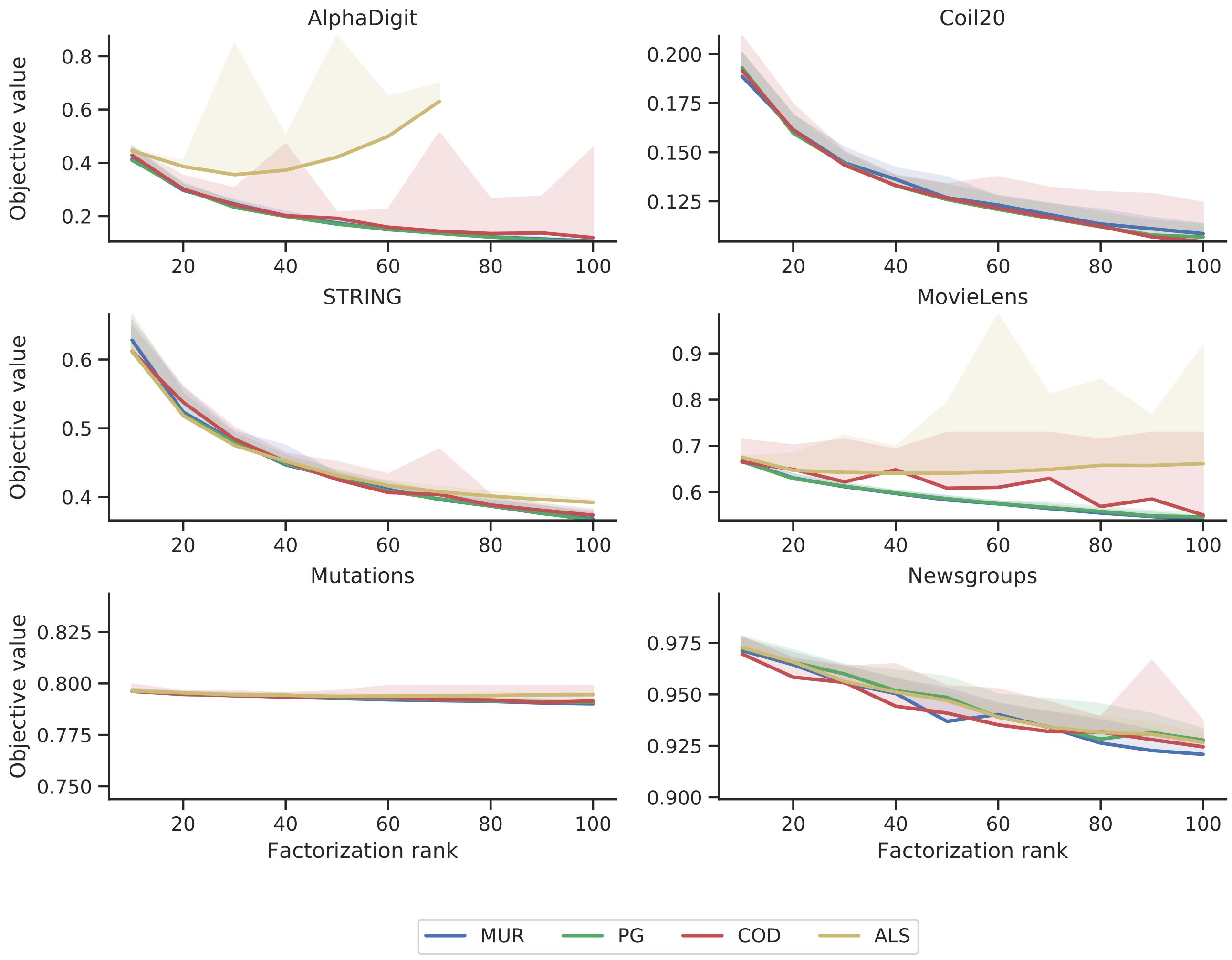

Figure B. Reconstruction of testing data $\mathbf{X}_{new}$ with respect to factorization rank. The lowest objective function at convergence is shown as a function of factorization rank ($k_1 = k_2$; $k_i \in \{10, 20, \ldots, 100\}$). The highlighted area shows the span from lowest to highest objective value across ten repeated runs. MUR, multiplicative update rules; ALS, alternating least squares; PG, projected gradients; COD, coordinate descent.

The convergence using pre-trained model is orders of magnitude faster than training from scratch (Fig. 2 in the main text). Consistent with the optimization on training data, we observe that Alternating least squares and Coordinate descent are fastest, Projected gradient is slower and Multiplicative updates is the slowest. ALS approach did not converge on Coil20 training data and was therefore not used on testing data.

Figure B shows the lowest objective value from multiple runs (solid lines). We compare the optimization function at convergence for factorization rank $k \in \{10, 20, \ldots, 100\}$. We can observe that for majority of datasets, all methods converge to a similar solution (Coil20, STRING, Mutations, Newsgroups). Coordinate descent and Alternating least squares appear more sensitive to random initialization on AlphaDigit and MovieLens datasets. The difference between best and worst solution increases as we increase factorization rank. In such cases, Coordinate descent needs to be repeated a few times in order to ensure results comparable to Projected gradients and Multiplicative update rules. Alternatively, using a different initialization technique[2] we may overcome this drawback.

G Stochastic mini-batch updates

We have implemented the stochastic mini-batch approach for NMTF. In each iteration we randomly permute the dataset into b batches such that each row is included in exactly one batch. We iterate over all batches and update the dataset using a subset of

the data $\mathbf{X}_i$ and a subset of $\mathbf{U}$ factor: $\mathbf{U}_i$, where $i \in \{1, 2, \ldots, b\}$. We split the data into $b = 20$ batches. The objective function is evaluated over the entire dataset.

We show the convergence of mini-batch versions together with its non-batch counterparts in Figure C. Each experiment was run for a maximum of 1000 iterations. Mini-batch versions of ALS (dashed yellow line) and COD (dashed red line) do not converge. Projected gradient mini-batch (dashed green line) and MUR mini-batch (dashed blue line) approaches show faster convergence at the beginning of some experiments (AlphaDigit, Coil20, MovieLens). The final solution is in all cases worse than the non-batch counterpart and large oscillations (larger than $\varepsilon = 0.01$) make it difficult to determine stopping criteria.

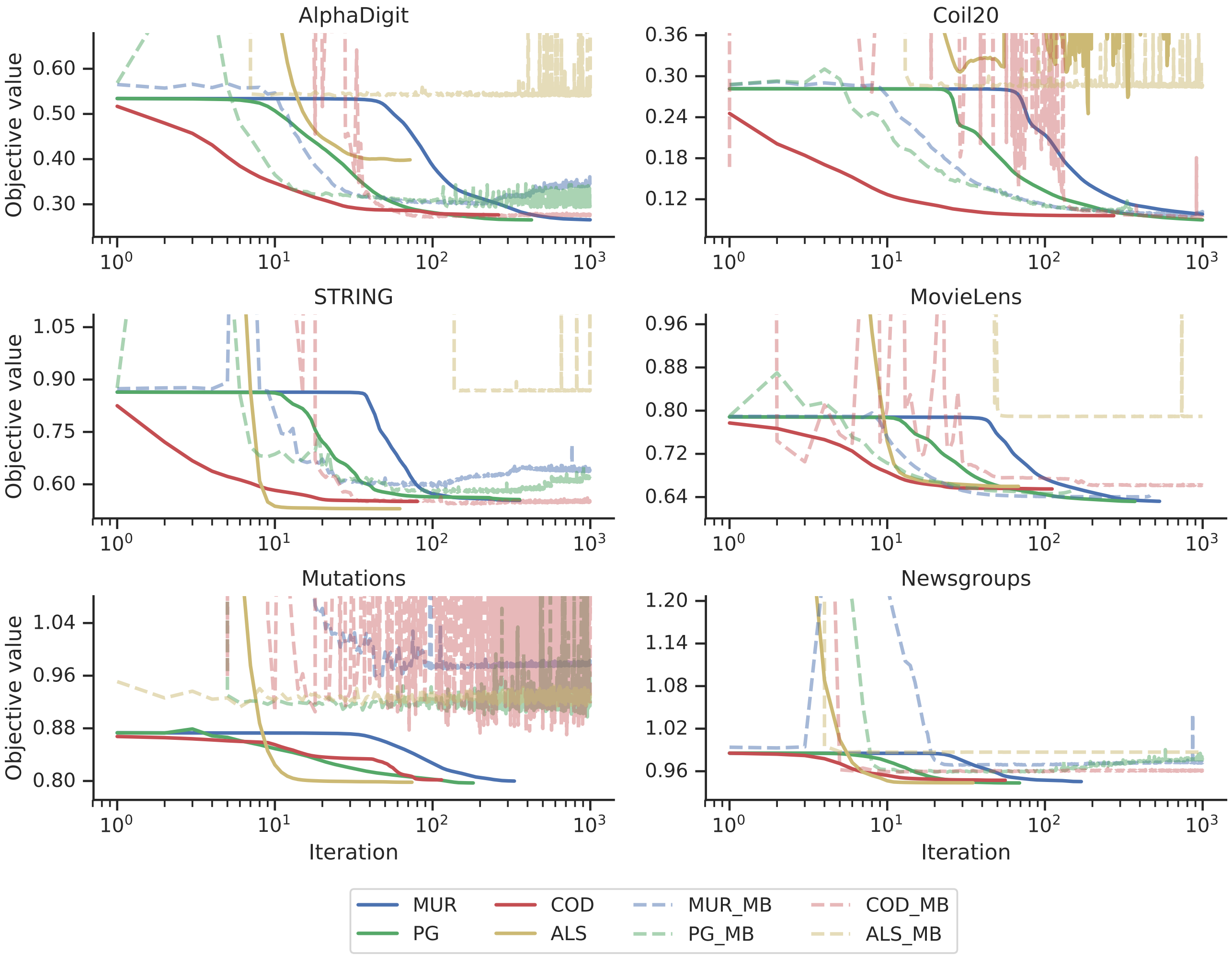

Figure C. Convergence of mini-batch optimization techniques on six datasets using four NMTF optimization methods (continuous lines) and four mini-batch variants (dashed transparent lines). MUR_MB: mini-batch multiplicative updates; PG_MB: mini-batch projected gradients; COD_MB: mini-batch coordinate descent; ALS_MB: mini-batch ALS.

References

1. Cichocki A, Zdunek R, Phan AH, Amari Si. Nonnegative matrix and tensor factorizations: applications to exploratory multi-way data analysis and blind source separation. John Wiley & Sons; 2009.
2. Langville, A., Meyer, C., Albright, R. Initializations for the nonnegative matrix factorization. *KDD 2006 Philadelphia, PA USA* **12** (2006).
